# Improvement in activities of daily living in postoperative patients with cervical spondylotic myelopathy undergoing intensive rehabilitation in a convalescent rehabilitation ward: A retrospective observational study

**DOI:** 10.1097/MD.0000000000049252

**Published:** 2026-06-12

**Authors:** Shuichi Sasaki, Wataru Saito, Toshiyuki Nakazawa, Naoto Kamide, Takuya Maeda, Akari Kobayashi, Michinari Miyazaki, Tetsuharu Nakazono, Ryota Mihira, Takayuki Imura, Masayuki Miyagi, Yoko Masaki, Tomonori Kenmoku, Michinari Fukuda, Gen Inoue, Masashi Takaso

**Affiliations:** a Department of Rehabilitation, Kitasato University Hospital, Sagamihara, Kanagawa, Japan; b Department of Orthopaedic Surgery, Kitasato University Medical Center, Kitamoto, Japan; c Department of Orthopaedic Surgery, Kitasato University School of Medicine, Sagamihara, Japan; d Department of Rehabilitation, School of Allied Health Sciences, Kitasato University, Sagamihara, Japan.

**Keywords:** activities of daily living, cervical spondylotic myelopathy, functional independence measure, functional prognosis, rehabilitation

## Abstract

Cervical spondylotic myelopathy (CSM) often leads to persistent impairment in motor function and activities of daily living (ADL) even after surgical decompression. Although postoperative rehabilitation is widely implemented, factors associated with ADL outcomes during the convalescent rehabilitation phase remain unclear. This study aimed to investigate ADL outcomes and their associated factors in postoperative patients with CSM admitted to a convalescent rehabilitation ward. This retrospective observational study included postoperative patients with CSM admitted to a convalescent rehabilitation ward. All patients underwent intensive multidisciplinary rehabilitation. ADL outcomes were assessed using the functional independence measure (FIM). Associations between ADL outcomes, disease severity, and rehabilitation-related factors were analyzed. At discharge, 91.2% of patients achieved independent transfer function, and 97.1% were discharged home. Although motor FIM scores at discharge remained lower in the severe group, greater improvement in motor FIM scores was associated with longer rehabilitation duration, particularly in patients with severe impairment. Most postoperative patients with CSM achieved favorable ADL outcomes during the convalescent rehabilitation phase. The findings suggest that the duration of intensive rehabilitation may be an important factor associated with motor function improvement after surgery for CSM, particularly in patients with severe impairment.

## 1. Introduction

In Japan, convalescent rehabilitation wards provide intensive multidisciplinary rehabilitation for patients after the acute phase of illness or surgery, with the aim of improving activities of daily living (ADL) and facilitating discharge to home.

As a discharge criterion, improvement in motor functional independence measure (FIM) scores, particularly transfer scores, is considered important for discharge to home.^[1,2]^ Diseases commonly treated in convalescent rehabilitation wards include cervical spondylotic myelopathy (CSM), a degenerative cervical spinal cord disorder associated with impaired hand dexterity and gait disturbances caused by cervical cord compression.^[3–6]^ Although surgical decompression is effective for preventing further neurological deterioration, many patients continue to experience postoperative impairments in gait, hand function, and ADL. Therefore, postoperative rehabilitation plays an important role in maximizing functional recovery and facilitating discharge to home.

The convalescent rehabilitation ward in our hospital treats patients who have undergone surgery for CSM and patients with severe impairment of motor function and ADL on admission. However, we have observed that some patients who required full assistance for ADL on admission later became mostly independent in ADL and were able to walk independently by the time of discharge. These observations suggest that early prediction of functional recovery after intensive rehabilitation may be difficult in patients with severe postoperative impairment. In patients with traumatic spinal cord injuries, upper limb recovery can be evaluated using manual muscle testing, and previous studies have shown that approximately 80% of upper limbs with scores of 1 to 2 on admission improved to scores of ≥3 within 3 to 6 months, regardless of functional level or surgical status.^[7,8]^ ADL and gait function reportedly recover significantly within the first 6 months after surgery, followed by stabilization over the subsequent year. While upper limb function often recovers relatively early, gait function tends to improve more gradually, highlighting the critical importance of rehabilitation.^[9]^ Given such considerations, predicting the recovery of lower limb function – including transfer ability and gait function – is essential for convalescent rehabilitation wards to optimize rehabilitation strategies and facilitate patient independence. However, little is known about the recovery process and functional prognosis of postoperative patients with CSM undergoing intensive rehabilitation in convalescent rehabilitation wards, particularly among patients with severe functional impairment. Therefore, the present retrospective observational study was conducted to investigate ADL outcomes and factors associated with functional improvement in postoperative patients with CSM undergoing intensive rehabilitation in a convalescent rehabilitation ward.

## 2. Materials and methods

This single-center retrospective observational study included 34 consecutive patients (23 men, 11 women; mean age, 70.9 years) who underwent cervical decompression surgery and were hospitalized in the convalescent rehabilitation ward of our hospital between July 2015 and December 2017. Patients with ossification of the posterior longitudinal ligament were excluded from the study. ADL severity and improvement were evaluated using the FIM. The data evaluated included age, sex, body mass index, operative methods, postoperative complications (presence or absence of pneumonia or surgical site infection), interval between symptom onset and surgery, length of stay in acute-care hospitals, length of stay in the convalescent rehabilitation ward, surgical procedure, anterior/posterior decompression and fixation, spinal cord level, preoperative modified Japanese Orthopaedic Association (mJOA) score, grip strength on admission, deltoid strength on admission, disturbance of deep sensation (presence or absence of proprioceptive impairment of the thumb), cognitive FIM scores on admission, motor FIM scores on admission and at discharge, and destination after discharge. The primary outcome was the change in motor FIM score (ΔFIM motor score) from admission to discharge. Secondary outcomes included discharge destination and independence in transfer function at discharge.

All patients underwent intensive multidisciplinary rehabilitation in the convalescent rehabilitation ward. Rehabilitation was provided 7 days per week for a total of 2 hours per day, consisting of 1 hour of physical therapy and 1 hour of occupational therapy. Physical therapy included gait training, balance training, muscle strengthening exercises, and outdoor walking training. Occupational therapy included upper limb functional training, such as muscle strengthening, fine motor skill training, ADL training, and instrumental ADL training. The rehabilitation program was based on a task-oriented approach and was tailored to each patient’s functional status and goals. Details of the rehabilitation program are summarized in Table 1.

**Table 1:** Details of the intensive rehabilitation program in the convalescent rehabilitation ward.

Component	Description
Frequency	7 d/wk
Daily duration	2 hr/d (1 hr PT and 1 hr OT)
PT	Gait training, balance training, muscle strengthening exercises, outdoor walking training
OT	Upper limb functional training, including muscle strengthening, fine motor skill training, ADL training, and instrumental ADL training
Rehabilitation approach	Task-oriented training tailored to each patient’s functional status and goals
Setting	Convalescent rehabilitation ward with multidisciplinary care

The rehabilitation program was structured and individualized according to each patient’s condition, including range-of-motion exercises, muscle strengthening, gait training, and ADL training. Therapy was provided daily, and the duration and intensity were adjusted based on patient tolerance and recovery status.

ADL = activities of daily living, FIM = functional independence measure, OT = occupational therapy, PT = physical therapy.

In this study, patients with an FIM score <67 on admission were classified as the severe group, whereas those with scores ≥67 were classified as the mild group. Previous studies have established a cutoff value of 63 to 64 points for the FIM motor score as a reliable predictor of home discharge.^[10]^ In the present study, we adopted a more stringent threshold of 67 points, reflecting its enhanced clinical applicability. This revised cutoff is consistent with the findings of Weinrebe et al,^[11]^ who identified 67 points as a critical benchmark for home discharge, suggesting that patients falling below this threshold are at a significantly higher risk of requiring institutional care or prolonged assistance.

For statistical analyses, comparisons between severe and mild groups were performed using an independent *t* test for continuous variables and the chi-squared test for categorical variables. A *P* value <.05 was considered statistically significant. The change in motor FIM score (ΔFIM motor score) was calculated as the difference between discharge and admission scores. A general linear model was used to evaluate the association between ΔFIM motor score and rehabilitation duration, adjusting for age and sex. A multivariable analysis was performed to adjust for potential confounding factors. Variables with *P* < .10 in univariate analyses were entered into the multivariable model. Multicollinearity was assessed using variance inflation factors.

This study was conducted in accordance with the ethical principles of the Declaration of Helsinki and was approved by the Kitasato University Institutional Review Board (approval no. B21-147). Due to the retrospective nature of the study using existing medical records, the requirement for informed consent was waived by the institutional review board. Information about the study and the opportunity to opt out were provided on the institutional website.

## 3. Results

Eighteen patients were assigned to the severe group and 16 patients to the mild group based on FIM motor scores on admission. Regarding transfer function at discharge, 31 of the 34 patients (91.2%) achieved independence and a score of ≥6 in the FIM transfer category. In terms of destination after discharge, 33 of the 34 patients were discharged home, and the remaining patient was transferred to a nursing home. The following evaluation categories showed significant differences between groups: age (severe group, 75.4 ± 8.6 years; mild group, 65.9 ± 10.4 years [*P* = .006]), sex (severe group, 44% males; mild group, 93% males [*P* = .002]), postoperative complications (severe group, 33%; mild group, 0% [*P* = .011]), length of stay in the convalescent rehabilitation ward (severe group, 88.3 ± 44.3 days; mild group, 45.1 ± 26.8 days [*P* = .002]), mJOA score (severe group, 6.5 ± 3.1; mild group, 11.8 ± 2.1 [*P* = .001]), grip strength on admission (severe group, 14.4 ± 7.7 kg; mild group, 25.2 ± 12.5 kg [*P* = .004]), disturbance of deep sensation (severe group, 56%; mild group, 13% [*P* = .009]), FIM motor score on admission (severe group, 44.3 ± 18.2; mild group, 77 ± 6.8 [*P* = .001]), and FIM motor score at discharge (severe group, 75.6 ± 10.5; mild group, 85.2 ± 3.7 [*P* = .001]; Table 2).

**Table 2:** Patient characteristics.

Assessment/measurement items	FIM motor score < 67 (severe, n = 18)	FIM motor score ≥ 67 (mild, n = 16)	*P* value
Sex (male/female)	8/10	15/1	.002**
Age (yr)	75.4 ± 8.6 (54–93)	65.9 ± 10.4 (45–83)	.006**
BMI (kg/m^2^)	23.3 ± 4.7	25.7 ± 4.2	.1237
Acute hospitalization period (d)	22.0 ± 11.5	15.7 ± 4.1	.04*
Rehabilitation hospitalization period (d)	88.3 ± 44.3 (39–150)	45.1 ± 26.8 (16–97)	.002**
Duration from symptom onset to surgery (d)	213.3 ± 208.5	206.2 ± 228.5	.9255
Surgical procedure (laminoplasty – open door/split)	7/9	3/11	.236
Anterior/posterior decompression and fixation	1/1	1/1	.618
Spinal cord level (C3–6/C3–7/C4–6/C4–7/C5–7/C6–7/up to T2)	5/4/3/1/2/3	8/5/0/1/0/2	.0315
Preoperative modified Japanese Orthopaedic Association	6.5 ± 3.1 (1.5–13.5)	11.8 ± 2.1 (7.5–15.0)	.001**
Rehabilitation hospitalization (d)	88.3 ± 44.3	45.1 ± 26.8	.002**
Discharge destination (home/facility)	17/1	16/0	.339
Grip strength at admission (right, kgf)	14.4 ± 7.7	25.2 ± 12.5	.004**
Grip strength at admission (left, kgf)	12.3 ± 7.5	24.5 ± 8.5	.001**
Deltoid MMT at admission (right)	4.0 ± 1.1	4.5 ± 0.8	.1369
Deltoid MMT at admission (left)	4.2 ± 0.9	4.6 ± 0.6	.1342
Presence of deep sensory impairment (yes/no)	10/8	2/14	.009**
Presence of postoperative complications (yes/no)	6/12	0/16	.011*
FIM motor score at admission	44.3 ± 18.2 (13–65)	77.0 ± 6.8 (77–91)	.001**
FIM cognitive score at admission	31.9 ± 5.7	34.6 ± 1.8	.07
Total FIM score at admission	76.2 ± 21.0	111.6 ± 6.8	.001**
FIM motor score at discharge	75.6 ± 10.5	85.2 ± 3.7	.001**

BMI = body mass index, FIM = functional independence measure, MMT = manual muscle testing.

Multiple regression analysis identified rehabilitation duration and FIM motor score on admission as factors associated with ΔFIM motor score (FIM < 67 = 0; FIM ≥ 67 = 1). The mild group showed a smaller improvement in FIM motor score than the severe group. Longer rehabilitation duration was significantly associated with greater improvement in motor FIM scores (Table 3).

**Table 3:** Factors associated with change in FIM from admission to discharge.

	Unstandardized coefficients	Standardized coefficients	Standard error	*T* value	*P* value
Intercept	7.485		14.97	0.5	.621
Age (yr)	0.136	0.09	0.197	0.694	.493
Sex (male)	3.614	0.108	4.374	0.826	.415
Intensive rehabilitation intervention period (d)	0.13	0.346	0.05	2.597	.015
Severity of motor FIM at admission (mild)	−14.407	−0.458	4.873	−2.956	.006

Adjusted *R*^2^: 0.600. Analysis of variance (ANOVA): *P* < .001.

FIM = functional independence measure.

**Figure 1.**
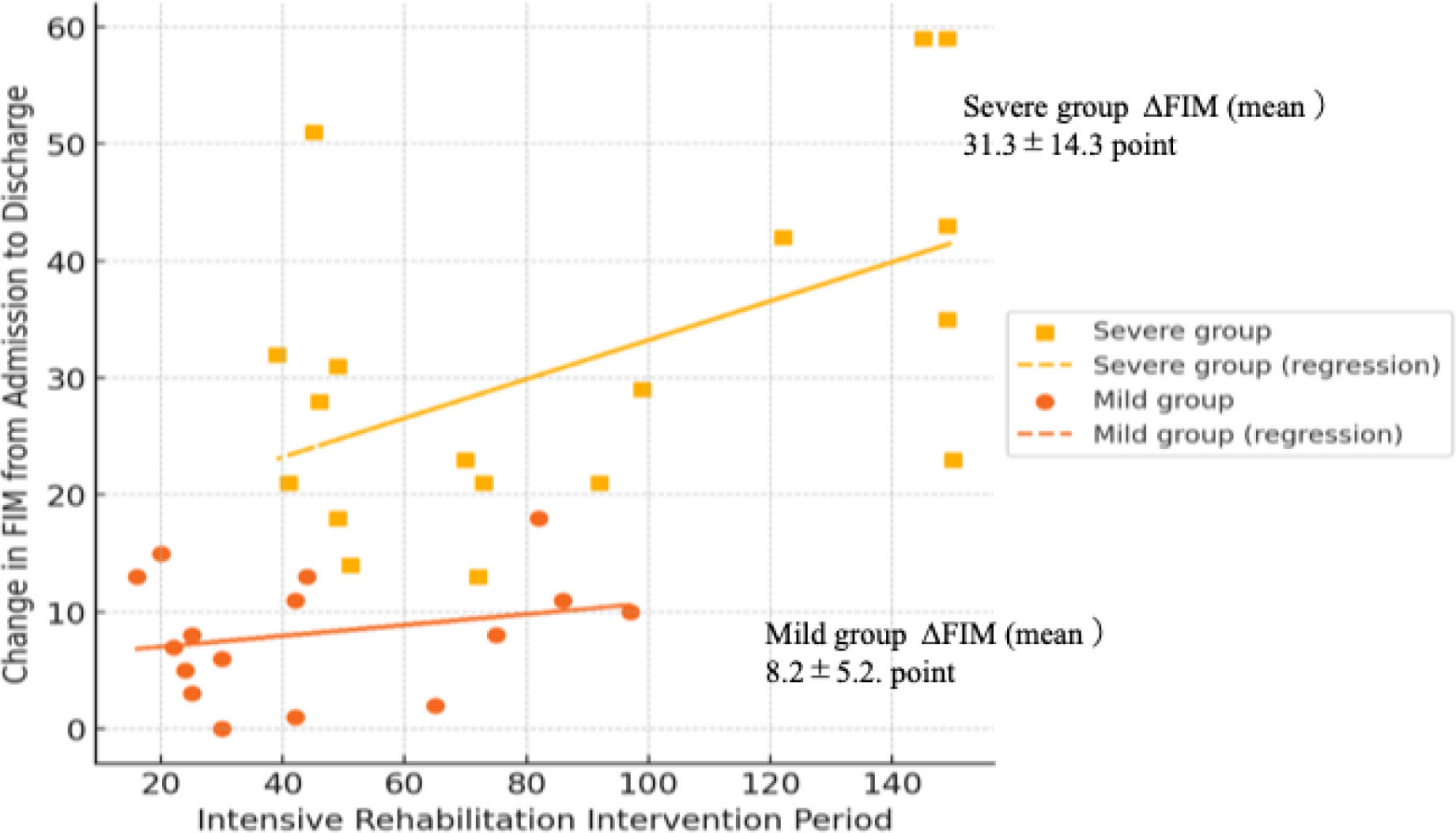
Correlation between FIM motor improvement and intensive rehabilitation period. A scatter plot illustrates the correlation between FIM motor improvement and the intensive rehabilitation period. In the severe group, a moderate positive correlation was observed (*r* = 0.514, *P* = .029), indicating that motor FIM improvement was significantly associated with rehabilitation duration. In contrast, the mild group exhibited a weak and non-significant correlation (*r* = 0.238, *P* = .375). FIM = functional independence measure.

Scatter plot analysis demonstrated a correlation between FIM motor improvement and rehabilitation duration (Fig. 1). The severe group showed a moderate positive correlation, Pearson correlation coefficient (*r*) = 0.514, *P* = .029, while the mild group showed a weak, non-significant correlation (*r* = 0.238, *P* = .375).

## 4. Discussion

This single-center retrospective observational study evaluated the functional prognosis of patients with CSM admitted to a convalescent rehabilitation ward. The present study demonstrated that motor FIM scores at discharge were lower in the severe group than in the mild group. However, the degree of improvement in motor FIM score during hospitalization was significantly associated with the duration of intensive rehabilitation, particularly in patients with severe impairment. These findings suggest that patients with severe motor impairment at admission may achieve greater functional improvement during the convalescent rehabilitation phase. Furthermore, the duration of hospitalization was longer in the severe group than in the mild group, suggesting that a longer duration of intensive rehabilitation was associated with greater improvement in ADL scores in the severe group. These findings suggest that even severely affected patients with CSM may achieve long-term functional improvement during the convalescent rehabilitation phase. This may reflect the characteristics of Japan’s convalescent rehabilitation wards, where patients receive 1 to 2 hours of rehabilitation daily.

Previous studies have identified younger age and higher preoperative mJOA score as significant predictors of favorable postoperative recovery.^[12,13]^ In addition, patients with symptom duration of <12 months tend to achieve better recovery of motor function post-surgery.^[14,15]^ The presence of postoperative complications has also been associated with limited recovery of motor function, particularly among elderly patients and those with multiple comorbidities.^[16]^

In this study, no significant difference in duration from symptom onset to surgery was observed. However, compared with the mild group, patients in the severe group showed significantly lower preoperative mJOA scores, were older at admission, and had a greater number of comorbidities. Nevertheless, by discharge, motor function in ADL had improved, and although a difference between groups remained evident, the severe group demonstrated a greater degree of improvement than the mild group. These findings suggest that sustained postoperative rehabilitation may contribute to functional recovery and improved quality of life. These findings may also provide valuable insights into clinical treatment planning and patient education. In addition, the quantity and quality of rehabilitation provided in convalescent rehabilitation wards may also contribute to functional recovery. This may include sufficient therapeutic exercise and task-oriented training aimed at improving ADL required for home discharge. Previous reports have highlighted rapid improvements in ADL and walking ability within the first 6 months post-surgery, followed by stabilization over the subsequent year.^[17]^ Motor function of the upper extremity tends to improve early in the postoperative period, significantly impacting ADL.^[15]^ Conversely, the likelihood of attaining complete recovery of gait function appears limited, particularly when the degree of improvement in lower extremity muscle strength is inadequate.^[16]^ However, some studies have shown significant improvements in walking ability and static balance at 3 and 6 months postoperatively, even among elderly patients, indicating that motor function can recover regardless of age. Our study demonstrated that, even in severe cases, patients were able to achieve independent ambulation and return home after extended periods of rehabilitation in a convalescent rehabilitation ward. These results suggest that sustained rehabilitation may be associated with functional recovery in patients with CSM.

A study on discharge to home in patients with cerebrovascular disorders demonstrated that rates of discharge to home were decreased in patients with severe impairment when performing ADL and a poor degree of independence in transfer function or in elderly patients.^[18,19]^ In addition, the study suggested that elderly patients with CSM showing severe impairment of ADL may achieve greater improvement in ADL performance compared with patients with cerebrovascular disorders. Although some reports have examined factors influencing the length of stay in acute-care hospitals,^[20,21]^ studies specifically addressing the duration of intensive functional recovery in a convalescent rehabilitation ward required for discharge to home are scarce. Functional impairments in patients with cerebrovascular disorders are often considered difficult to reverse once established. However, functional impairments in CSM patients are not the same as those in patients with cerebrovascular disorders, suggesting that improvement remains possible in the former group. To the best of our knowledge, few studies have examined the functional recovery process of postoperative patients with CSM undergoing intensive rehabilitation in convalescent rehabilitation wards. Our findings suggest that the average length of stay in a convalescent rehabilitation ward is approximately 3 months. When including the length of stay in acute-care hospitals, a total period of 3 to 6 months may be sufficient to facilitate home discharge even in patients with severe impairment.

Several limitations of this study should be acknowledged. Because this was a retrospective observational study without a control group, causal relationships could not be established. As a retrospective study, selection bias may have been present. The use of FIM alone may not capture subtle functional changes. First, the sample size was relatively small. Second, not all confounders were adjusted for in the multivariable analysis. The ADL status of patients prior to surgery was not assessed, so we could not clarify the effects of preoperative function on postoperative recovery of function. Further studies with larger sample sizes are warranted to examine the status of ADL before surgery. In addition, although the rehabilitation program was delivered within a standardized institutional framework, the specific content was individualized according to each patient’s functional status and goals. Therefore, the effects of individual rehabilitation components could not be isolated.

In conclusion, this study evaluated the functional prognosis of patients with CSM undergoing intensive rehabilitation in a convalescent rehabilitation ward. The findings suggest that prolonged intensive rehabilitation is significantly associated with motor function improvement, particularly among patients with severe ADL impairment on admission. Although the severe group showed lower motor FIM scores at discharge than the mild group, they also exhibited a greater degree of improvement during hospitalization. These findings suggest the potential importance of sustained rehabilitation interventions in functional recovery.

Furthermore, the present results suggest that a total hospitalization period of 3 to 6 months, including both acute care and rehabilitation, may be required to facilitate home discharge, even in patients with severe impairments. Future studies with larger sample sizes and assessments of preoperative ADL are needed to refine rehabilitation strategies and improve early functional prognosis. The present findings provide valuable insights for optimizing rehabilitation planning and enhancing patient outcomes.

## Acknowledgments

We would like to express our gratitude to Y.H., K.I., M.S., and C.M. for their cooperation in conducting this study. We also extend our sincere appreciation to the occupational therapists and physical therapists of the Rehabilitation Department at Kitasato East Hospital for their valuable support in this research.

## Author contributions

**Conceptualization:** Shuichi Sasaki, Wataru Saito, Toshiyuki Nakazawa, Naoto Kamide, Takuya Maeda, Michinari Miyazaki, Takayuki Imura, Tomonori Kenmoku, Michinari Fukuda, Masashi Takaso.

**Data curation:** Shuichi Sasaki, Naoto Kamide, Tetsuharu Nakazono.

**Formal analysis:** Shuichi Sasaki, Takayuki Imura, Masayuki Miyagi, Gen Inoue.

**Investigation:** Shuichi Sasaki, Takuya Maeda, Akari Kobayashi, Michinari Miyazaki, Tetsuharu Nakazono, Ryota Mihira, Yoko Masaki.

**Methodology:** Shuichi Sasaki, Wataru Saito, Takuya Maeda, Michinari Miyazaki, Tetsuharu Nakazono, Ryota Mihira, Yoko Masaki, Michinari Fukuda.

**Validation:** Shuichi Sasaki, Wataru Saito, Naoto Kamide, Takuya Maeda, Akari Kobayashi, Tetsuharu Nakazono, Ryota Mihira, Masayuki Miyagi, Yoko Masaki, Tomonori Kenmoku, Gen Inoue.

**Visualization:** Shuichi Sasaki, Akari Kobayashi, Michinari Miyazaki.

**Project administration:** Wataru Saito, Toshiyuki Nakazawa, Naoto Kamide, Tomonori Kenmoku, Michinari Fukuda, Gen Inoue, Masashi Takaso.

**Supervision:** Wataru Saito, Toshiyuki Nakazawa, Naoto Kamide, Masayuki Miyagi, Yoko Masaki, Michinari Fukuda, Masashi Takaso.

**Resources:** Toshiyuki Nakazawa, Takayuki Imura, Masayuki Miyagi, Michinari Fukuda.

**Writing – original draft:** Shuichi Sasaki, Naoto Kamide.

**Writing – review & editing:** Shuichi Sasaki, Wataru Saito, Toshiyuki Nakazawa, Takuya Maeda, Akari Kobayashi, Michinari Miyazaki, Tetsuharu Nakazono, Ryota Mihira, Takayuki Imura, Masayuki Miyagi, Yoko Masaki, Tomonori Kenmoku, Michinari Fukuda, Gen Inoue, Masashi Takaso.
